# Active inference and robot control: a case study

**DOI:** 10.1098/rsif.2016.0616

**Published:** 2016-09

**Authors:** Léo Pio-Lopez, Ange Nizard, Karl Friston, Giovanni Pezzulo

**Affiliations:** 1 Pascal Institute, Clermont University, Clermont-Ferrand, France; 2 Institute of Cognitive Sciences and Technologies, National Research Council, Rome, Italy; 3 The Wellcome Trust Centre for Neuroimaging, Institute of Neurology, University College London, London, UK

**Keywords:** active inference, free energy, robot control

## Abstract

Active inference is a general framework for perception and action that is gaining prominence in computational and systems neuroscience but is less known outside these fields. Here, we discuss a proof-of-principle implementation of the active inference scheme for the control or the 7-DoF arm of a (simulated) PR2 robot. By manipulating visual and proprioceptive noise levels, we show under which conditions robot control under the active inference scheme is accurate. Besides accurate control, our analysis of the internal system dynamics (e.g. the dynamics of the hidden states that are inferred during the inference) sheds light on key aspects of the framework such as the quintessentially multimodal nature of control and the differential roles of proprioception and vision. In the discussion, we consider the potential importance of being able to implement active inference in robots. In particular, we briefly review the opportunities for modelling psychophysiological phenomena such as sensory attenuation and related failures of gain control, of the sort seen in Parkinson's disease. We also consider the fundamental difference between active inference and optimal control formulations, showing that in the former the heavy lifting shifts from solving a dynamical inverse problem to creating deep forward or generative models with dynamics, whose attracting sets prescribe desired behaviours.

## 1. Introduction

Active inference has recently acquired significant prominence in computational and systems neuroscience as a general theory of brain and behaviour [1,2]. This framework uses one single principle—surprise (or free energy) minimization—to explain perception and action. It has been applied to a variety of domains, which includes perception–action loops and perceptual learning [3,4]; Bayes optimal sensorimotor integration and predictive control [5]; action selection [6,7] and goal-directed behaviour [8–12].

Active inference starts from the fundaments of self-organization which suggests that any adaptive agent needs to maintain its biophysical states within limits, therefore maintaining a generalized homeostasis that enables it to resist the second law of thermodynamics [2]. To this aim, both an agent's actions and perceptions both need to minimize *surprise*, that is, a measure of discrepancy between the agent's current predictive or desired states. Crucially, agents cannot minimize surprise directly but they can minimize an upper bound of surprise, namely the free energy of their beliefs about the causes of sensory input [1,2].

This idea is cast in terms of Bayesian inference: the agent is endowed with priors that describe its desired states and a (hierarchical, generative) model of the world. It uses the model to generate continuous predictions that it tries to fulfil via action; that is to say, the agent activity samples the world to minimize prediction errors so that surprise (or its upper bound, free-energy) is suppressed. More formally, this is a process in which beliefs about (hidden or latent) states of the world maximize Bayesian model evidence of observations, while observations are sampled selectively to conform to the model [13,14]. The agent has essentially two ways to reduce surprise: change its beliefs or hypotheses (perception), or change the world (action). For example, if it believes that its arm is raised, but observes it is not, then it can either change its mind or to raise the arm—either way, its prediction comes true (and free energy is minimized). As we see, in active inference, this result can be obtained by endowing a Bayesian filtering scheme with reflex arcs that enable action, such as raising a robotic arm or using it to touch a target. In this example, the agent generates a (proprioceptive) prediction corresponding to the sensation of raising alarm, and reflex arcs fulfil this prediction effectively raising the hand (and minimizing surprise).

Active inference has clear relevance for robotic motor control. As in optimal motor control [15,16], it relies on optimality principles and (Bayesian) state estimation; however, it has some unique features such as the fact that it dispenses with inverse models (see the Discussion). Similar to planning-as-inference and KL control [17–22], it uses Bayesian inference, but it is based on the minimization of a free-energy functional that generalizes conventional cost or utility functions. Although the computations underlying Bayesian inference or free-energy minimization are generally hard, they become tractable as active inference uses *variational inference*, usually under the Laplace assumption, which enables one to summarize beliefs about hidden states with a single quantity (the conditional mean). The resulting (neural) code corresponds to the Laplace code, which is simple and efficient [3].

Despite its success in computational and systems neuroscience, active inference is less known in related domains such as motor control and robotics. For example, it remains unproven that the framework can be adopted in challenging robotic set-ups. In this article, we ask if active inference can be effectively used to control the 7-DoF arm of a PR2 robot (simulated using Robot Operating System (ROS)). We present a series of robot reaching simulations under various conditions (with or without noise on vision and/or proprioception), in order to test the feasibility of this computational scheme in robotics. Furthermore, by analysing the internal system dynamics (e.g. the dynamics of the hidden states that are inferred during the inference), our study sheds light on key aspects of the framework such as the quintessentially multimodal nature of control and the relative roles of proprioception and vision. Finally, besides providing a proof of principle for the usage of active inference in robotics, our simulations help to illustrate the differences between this scheme and alternative approaches in computational neuroscience and robotics, such as optimal control, and the significance of these differences from both a technological and biological perspective.

## 2. Methods

In this section, we first define, mathematically, the active inference framework (for the continuous case). We then describe its application to robotic control and reaching.

### 2.1. Active inference formalism

The free-energy term that is optimized (minimized) during action control rests on the tuple 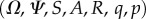 [23]. A real-valued random variable is denoted by 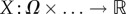 and 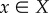 for a particular value. The tilde notation 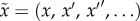 corresponds to variables in generalized coordinates of motion [24]. Each prime is a temporal derivative. *p*(*X*) denotes a probability density.

— *Ω* is the sample space from which random fluctuations 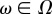 are drawn.
— Hidden states 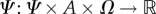. They depend on actions and are part of the dynamics of the world that causes sensory states.
— Sensory states 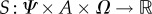. They are the agent's sensations and constitute a probabilistic mapping from action and hidden states.
— Action 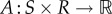. They are the agent's actions and depend on its sensory and internal states.
— Internal states 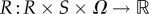. They depend on sensory states and cause actions. They constitute the dynamics of states of the agent.
— A recognition density 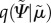, which corresponds to the agent's beliefs about the causes *Ψ* (and brain state *μ* describing those beliefs).
— A generative density 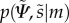 corresponding to the density of probabilities of the sensory states *s* and world states *Ψ*, knowing the predictive model *m* of the agent.

According to Ashby [25], in order to restrict themselves in a limited number of states, an agent must minimize the dispersion of its sensory and hidden states. The Shannon entropy corresponds to the dispersion of the external states (here *S* × *Ψ*). Under ergodic assumption, this entropy equals the long-term average of Gibbs energy2.1
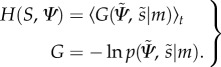
One can see that the Gibbs energy is defined in terms of the generative model. 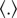 is the expectation or the mean under a density when indicated. However, agents cannot minimize this energy directly, because hidden states are unknown by definition.

However, mathematically2.2
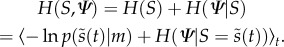


With this latter equation, we observe that sensory surprise 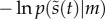 minimizes the entropy of the external states and can be minimized through action if action minimizes conditional entropy. In this sense2.3
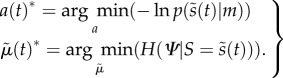


Unfortunately, we cannot minimize sensory surprise directly (see equation (2.3)) as this entails a marginalization over hidden states which is intractable2.4
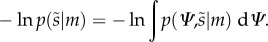


Happily, there is a solution to this problem that comes from theoretical physics [26] and machine learning [27] called variational free energy, which furnishes an upper bound on surprise. This is a functional of the conditional density, which minimized by action and internal states, to produce action and perception2.5
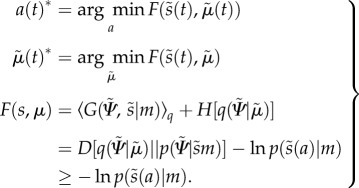


The term 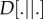 is the Kullback–Leibler divergence (or cross-entropy) between two densities. The minimizations on *a* and 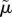 correspond to action and perception, respectively, where the internal states 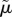 parametrize the conditional density *q*. We need perception in order to use free energy to finesse the (intractable) evaluation of surprise. The Kullback–Leibler term is non-negative, and the free energy is therefore always greater than surprise as we can see it in the last inequality. When free energy is minimized, it approximates surprise and as a result, the conditional density *q* approximates the posterior density over external states2.6
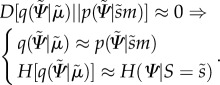


This completes a description of approximate Bayesian inference (active inference) within the variational framework. This free-energy formulation resolves several issues in perception and action control problems, but in the following, we focus on action control. According to equation (2.5), free energy can be minimized using actions via its effect on hidden states and sensation. In this case, action changes the sensations to match the agent's expectations.

The only outstanding issue is the nature of the generative model used to explain and sample sensations. In continuous time formulations, the generative model is usually expressed in terms of coupled (stochastic) differential equations. These equations describe the dynamics of the (hidden) states of the world and the ensuing behaviour of an agent [5]. This leads us to a discussion of the agent's generative model.

### 2.2. The generative model

Active inference generally assumes that the generative model supporting perception and action is nonlinear, dynamic and deep (i.e. hierarchical), of the sort that might be entailed by cortical and subcortical hierarchies in the brain [28].2.7
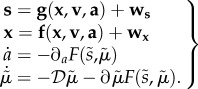


In bold, we have real-world states and in italic, internal states of the agent. **s** is the sensory input, **x** corresponds to hidden states, **v** to hidden causes of the world and **a** to action. Intuitively, hidden states and causes are used by the brain as abstract quantities in order to predict sensations. Dynamics over time is linked by hidden states, whereas the hierarchical levels are linked by hidden causes. The  notation means that we are using generalized coordinates of motion, i.e. a vector of positions, velocities, accelerations, etc. [5]. 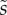, 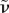 and *a* corresponds to sensory input, conditional expectations and action, respectively.

One can observe a coupling between these differential equations: sensory states depend upon action *a*(*t*) via causes (**x**, **v**) and the functions (**f**, **g**). While action depends upon sensory states via internal states 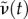. These differential equations are stochastic owing to random fluctuations (*ω_x_*, *ω_v_*).

A generalized gradient descent on variational free energy is defined in the second pair of equations. This method is termed *generalized filtering* and rests on conditional expectations to produce a prediction (first) term and an update (second) term based upon free-energy gradients that, as we see below, can be expressed in terms of prediction errors (this corresponds to the basic form of a Kalman filter). 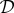 is a differential matrix operator that operates on generalized motion and 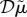 describes the generalized motion of conditional expectations. Generalized motion comprises vectors of velocity, acceleration, jerk, etc.

The generative model has the following hierarchical form2.8
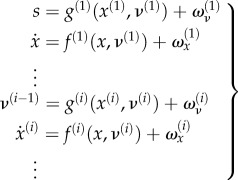


The level of the hierarchy in the generative model corresponds to *i*. *f*^(*i*)^ and *g*^(*i*)^ and their Gaussian random fluctuations *ω_x_* and *ω_v_* on the motion of hidden states and causes define a probability density over sensations, causes of the world and hidden states that constitute the free energy of posterior or conditional (Bayesian) beliefs about the causes of sensations. Note that the generative model becomes probabilistic because of the random fluctuations (where sensory or sensor noise corresponds to fluctuations at the first level of the hierarchy and at fluctuations at higher levels induces uncertainty about hidden states). The inverse of the covariances matrices of these random fluctuations is called precision (i.e. inverse covariance) and is denoted by 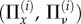.

### 2.3. Prediction errors and predictive coding

We can now define prediction errors on the hidden causes and states. These auxiliary variables represent the difference between conditional expectations and their predicted values based on the level above. Using 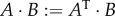:2.9
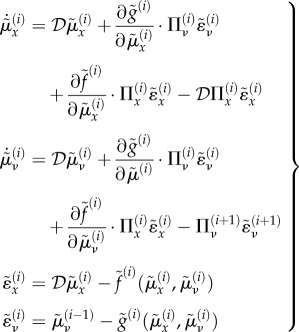



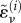
 and 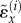 correspond to prediction errors on hidden causes and hidden states, respectively. The precisions 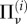 and 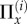 weights the prediction errors, so that more precise prediction errors have a greater influence during generalized filtering.

The derivation of equation (2.8) enables us to express the gradients equation (2.7) in terms of prediction errors. Effectively, precise prediction errors update the prediction to provide a Bayes optimal estimate of hidden states as a continuous function of time—where free energy corresponds to the sum of the squared prediction error (weighted by precision) at each level of the hierarchy. Heuristically, this corresponds to an instantaneous gradient ascent in which prediction errors are assimilated to provide for online inference. For a more detailed explanation of the mathematics under this scheme, see [24].

### 2.4. Action

A motor trajectory (e.g. the trajectory of raising the arm) is produced via classical reflex arcs that suppress proprioceptive prediction errors2.10
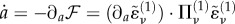


Intuitively, conditional expectations in the generative model drive (top-down) proprioceptive predictions (e.g. the proprioceptive sensation of raising one's own arm), and these predictions are fulfilled by reflex arcs. This is because the only way for an agent to minimize its free energy through action (and suppress proprioceptive prediction errors) is to change proprioceptive signals, i.e. raise the arm and realize the predicted proprioceptive sensations. According to this scheme, reflex arcs thus produce a motor trajectory (raising the arm) to comply with set points or trajectories prescribed by descending proprioceptive predictions (cf. motor commands). At the neurobiological level, this process is thought to occur at the level of cranial nerve nuclei and spinal cord.

### 2.5. Application to robotic arm control and reaching

**Figure 1.**
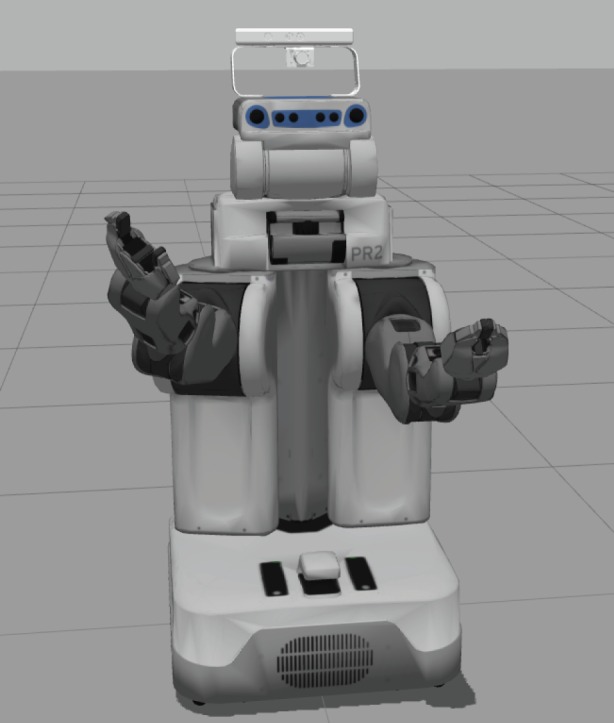
PR2 robot simulated using ROS [29].

Having described the general active inference formalism, we now illustrate how it can be used to elicit reaching movements with a robot: the 7-DoF arm of a PR2 robot simulated using the ROS [29] (figure 1). Essentially, in our simulations, the robot has to reach a target by moving (i.e. raising) its arm. We see that the key aspect of this behaviour rests on a multimodal integration of visual and proprioceptive signals [30,31], which play differential—yet interconnected—roles.

In this robotic setting, the hidden states are the angle of the joints 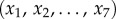. The visual input is the position of the end effector, here the arm of the PR2 robot. This location 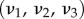 can be seen as autonomous causal states. We assume that the robot knows the true mapping between the position of its hand *Pos* and the angles of its joints. In other words, we assume that the robot knows its forward model and can extract the true position of its end effector in three-dimensional coordinates into the visual space.2.11
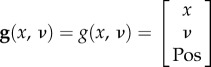


If we assume a Newtonian dynamics with viscosity *κ* and elasticity *k*, then we obtain the subsequent equations of motion that describe the true (physical) evolution of hidden states2.12
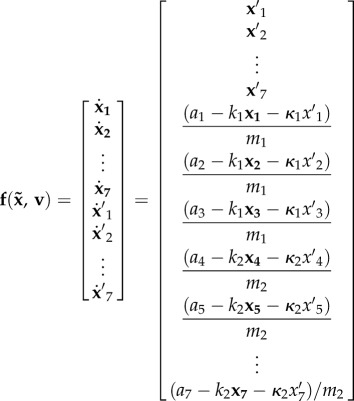


The behaviour of the robot arm during its reaching task is specified in terms of the robot's prior beliefs that constitute its generative model. Here, these beliefs are based upon a basic but efficient feedback control. In other words, by specifying a particular generative model, we create a robot that thinks it will behave in a particular way: in this instance, we think it behaves as an efficient feedback controller, as follows. Within the joint configuration space (thanks to geometrical considerations), the prior control law provides a per-joint angular increment to be applied according to the position of the end effector, allowing its convergence towards the target position. In order to avoid the singular configurations of the PR2 arm, two actions *α* and *β* are superposed. The first one is a per-joint action: each joint tries to align the portion of arm it supports with the target position. The second action is distributed over the shoulder, and the elbow providing the flexion–extension primitive in order to reach or escape the singular configurations of the first action (e.g. stretched arm).

Let *T* = (*t*_1_, *t*_2_, *t*_3_) be the target position in the Euclidean space, 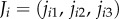 the position of the joint *i* in 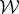, 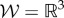, 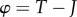 the vector describing the shortest path in 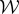 to reach the target, 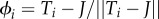 the unit vector linking each joint to the arm's distal extremity, 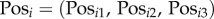 the unit vector collinear to the rotation axis of the joint *i*. Let ‘

’ be the dot product in 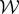 and ‘×’ the cross product. The feedback error to be regulated to zero by the first action of the control law for the joint *i* is2.13
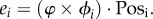


Classically, the first action is designed as a PI controller that ensures2.14
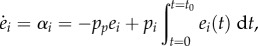
where *t*_0_ is the current time and {*p_p_*,*p_i_*} are two positive settings used to adjust the convergence rate. To preclude wind-up phenomena, the absolute value of the integral term is bounded by *α*_max_ > 0.

To operate as expected, the second action needs to predict the influence of the ‘stretched arm’ singularity. This is achieved with two parameters *γ_m_* and *γ_c_*. They are defined as the dot products2.15
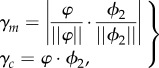
where the absolute value of *γ_c_* is bounded by *γ*_max_ > 0. Then, the second action is defined as2.16
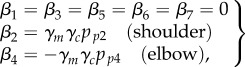
where 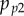 and 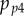 are additional positive settings used to balance the contribution of the two joints (roughly: 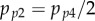). Finally, the controller provides the empirical prior2.17
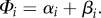


In practice, to obtain reasonable behaviour, the controller settings were chosen as: *p_p_* = 0.3, *p_i_* = 0.01, *α*_max_ = 0.001, *p_p_*_2_ = 2.25, *p_p_*_4_ = 5, *γ*_max_ = 0.1.

Finally, we obtain the following generative model2.18
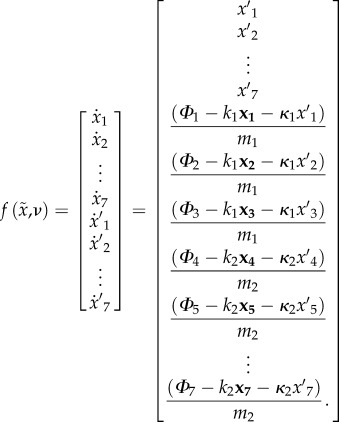


Importantly, we see that the generative model has a very different form from the true equations of motion. In other words, the generative model has prior beliefs that render motor behaviour purposeful. It is this enriched generative model that produces goal-directed behaviour, which fulfils the robot's predictions and minimizes surprise. In this instance, the agent believes it is going to move with its arm towards the target until it touches it. The distance between the end effector and the target is used as an error that drives the motion, as if the end effector is pulled to the target. The ensuing movement therefore resolves the Bernstein's problem that tries to solve the converse problem of pushing the end effector towards the target (which is an ill-posed problem). This formulation of motor control is related to the equilibrium point hypothesis [32] and the passive motion paradigm [33,34] and, crucially, dispenses with inverse models. Note that no solution of an optimal control problem is required here. This is because the causes of desired behaviour are specified explicitly by the generative or forward model (the arm is pulled to a target), and do not have to be inferred from desired consequences; see section Discussion for a comparison of active inference and optimal control schemes.

## 3. Results

**Figure 2.**
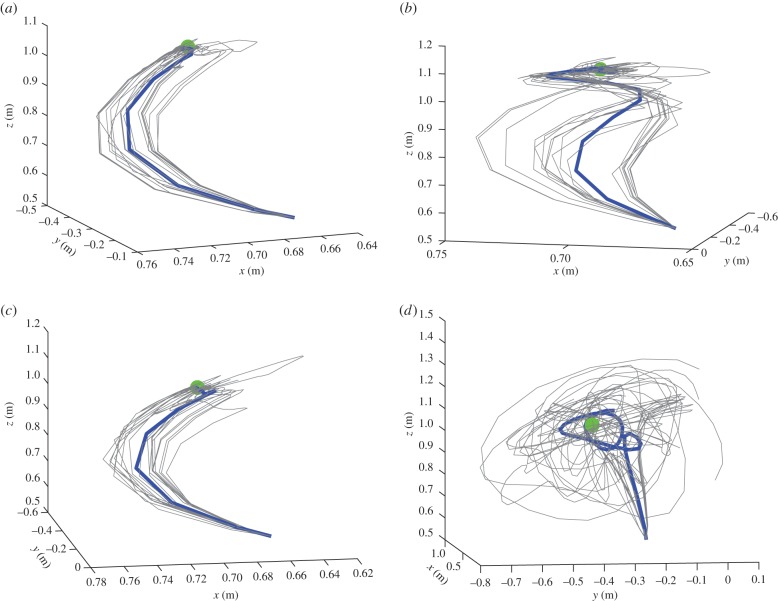
Reaching trajectories in three dimensions from a start to a goal location under four scenarios. (*a*) Scenario 1: reaching in three dimensions with 7 DoF. (*b*) Scenario 2: reaching in three dimensions with noisy proprioception. (*c*) Scenario 3: reaching in three dimensions with noisy vision. (*d*) Scenario 4: reaching in three dimensions with noisy proprioception and vision. The blue trajectory is the mean of 20 trajectories shown in grey.

We tested the model in four scenarios. In all the scenarios, the robot arm started from a fixed starting position and had to reach a desired position in three dimensions with its 7-DoF arm. We simulated various starting and desired positions, but in this illustration, we focus on the sample problem illustrated in figure 2, where the start position is on the bottom-centre, and the desired position is the green dot. The four panels of figure 2 exemplify the robot reaching under the four scenarios that we considered. In the first scenario (figure 2*a*), there was no noise on proprioception and vision. In the second, third and fourth scenarios, proprioception (figure 2*b*), vision (figure 2*c*) or both (figure 2*d*) were noisy, respectively. We used noise with a log precision of 4.

As illustrated by the figures, in the absence of noise (first scenario), the reaching trajectory is flawless and free of static error (figure 2*a*). Trajectories become less accurate when either proprioception (second scenario) or vision (third scenario) are noisy, still the arm reaches the desired target (figure 2*b*,*c*). However, when both proprioception and vision are noisy, the arm becomes largely unable to reach the target (figure 2*d*).

**Figure 3.**
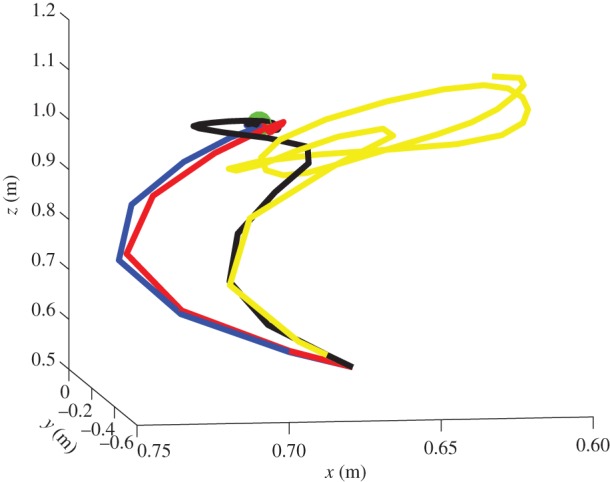
Reaching trajectories from a common starting point. In blue, no noise (scenario 1). In black, noise on proprioception (scenario 2). In red, noise on vision (scenario 3). In yellow, noise on vision and proprioception (scenario 4). The trajectories are the mean of 20 simulations from the same start and goal (green) locations.

A more direct comparison between the four scenarios is possible, if one considers the average of 20 simulations from a common starting point (figure 3). Here, the four colours correspond to the four scenarios: first scenario (no noise) is blue; second scenario (noisy proprioception) is black, third scenario (noisy vision) is red and fourth scenario (noisy proprioception and vision) is yellow. To compare the trajectories under the four scenarios quantitatively, we computed the sum of Euclidian distances between the position of the end effector for each iteration of the algorithm under the best trajectory (corresponding to scenario 1) and the other trajectories. We obtained a difference between the normal and noisy proprioception scenarios of 0.2796; between normal and noisy vision scenarios of 0.143; and a difference between the normal and noisy proprioception and vision scenarios of 1.2169.

**Figure 4.**
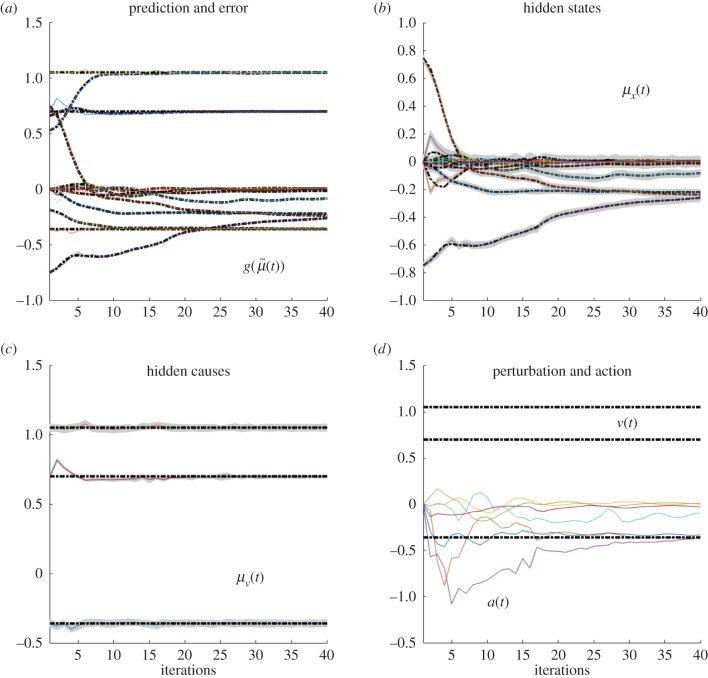
Dynamics of the model internal variables in the normal case. The conditional predictions and expectations are shown as functions of the iterations. (*a*) the panel shows the conditional predictions (coloured lines) and the corresponding prediction errors (red lines) based upon the expected states on the upper right. (*b*) The coloured lines represent the expected hidden states causing sensory predictions. These can be thought of displacements in the articulatory state space. In this panel and throughout, the grey areas denote 90% Bayesian confidence intervals. (*c*) The dotted lines represent the true expectation and the solid lines show the conditional expectation of the hidden cause. (*d*) Action (solid line) and true causes (dotted line).

**Figure 5.**
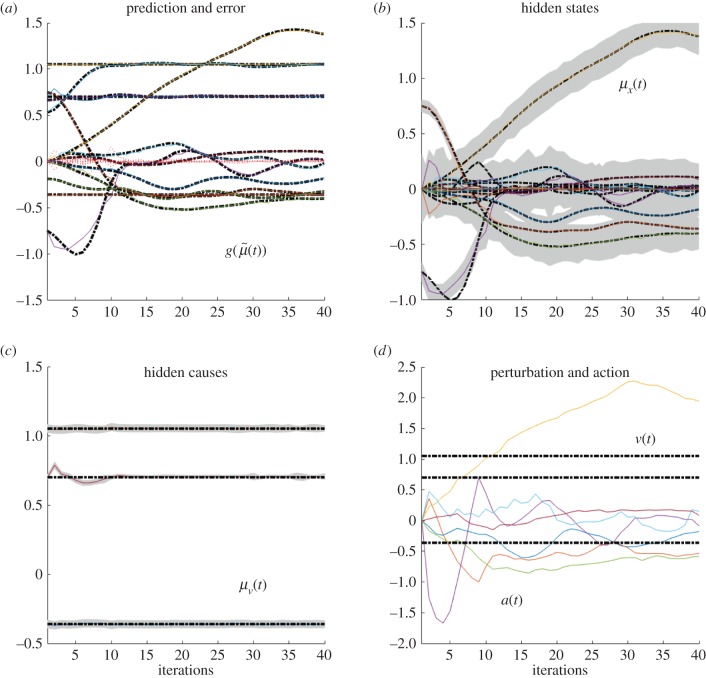
(*a*–*d*) Dynamics of the model internal variables in the noisy proprioception case. The layout of the figure is the same as figure 4. Please see the previous figure legends for details.

**Figure 6.**
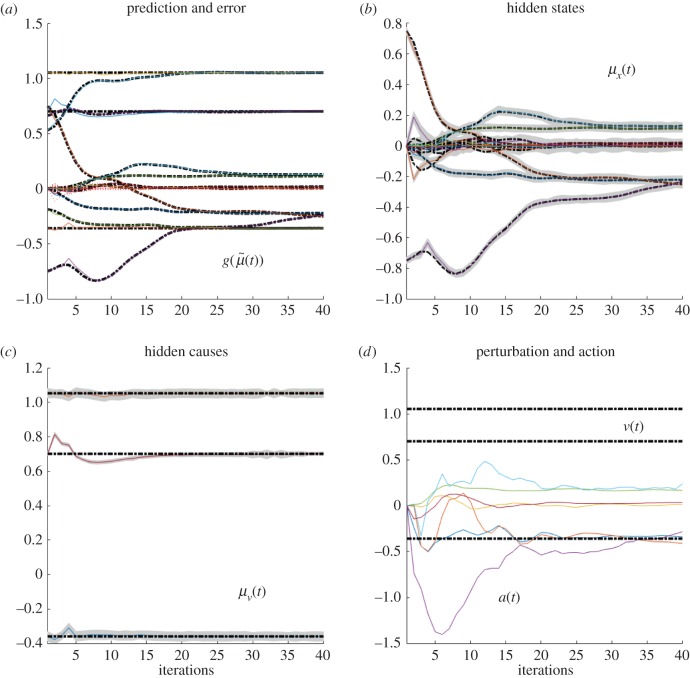
(*a*–*d*) Dynamics of the model internal variables in the noisy vision case. The layout of the figure is the same as figure 4. Please see the previous figure legends for details.

**Figure 7.**
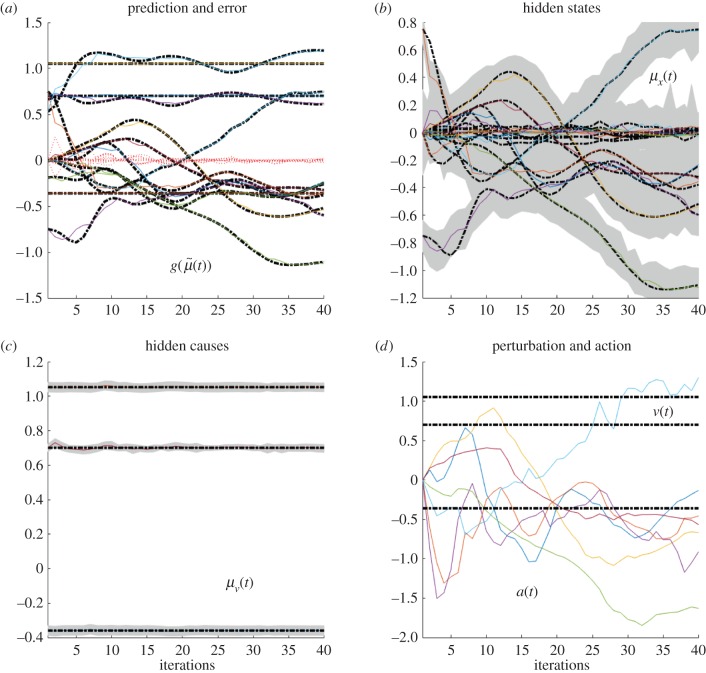
(*a*–*d*) Dynamics of the model internal variables in the ‘all noisy’ case. The layout of the figure is the same as figure 4. Please see the previous figure legends for details.

These differences can be better appreciated if one considers the internal dynamics of the system's hidden states (i.e. angles of the arm) during the different conditions, as shown in figures 4–7 for the simulations without noise, noisy proprioception, noisy vision and noisy proprioception and vision, respectively. The hidden states are inferred while the agent optimizes its expectations as described above (see equation (2.12)). In turn, action (*a*(*t*)) is selected based on the hidden states (technically, action is part of the generative process but not the generative model).

The four panels of figures 4–7 show the conditional predictions and prediction errors during the task. In each figure, the top right panel shows the hidden states, and the grey areas correspond to 90% Bayesian confidence intervals. The figures show that adding noise to proprioception (figure 5) makes the confidence interval much larger compared with a standard case with no noise (figure 4). Confidence intervals further increase when both proprioception and vision are noisy (figure 7). The top left panel shows the conditional predictions of sensory signals (coloured lines) and sensory prediction errors (red). These are errors on the proprioceptive and visual input, and are small in relation to predictions. The bottom left panel shows the true expectation (dotted line) and conditional expectation (solid line) about hidden causes. The bottom right panel shows actions (coloured lines) and true causes (dotted lines). In the noisy proprioception scenario (figure 5), one of the hidden states (top right panel) and one action (bottom right panel) rises with time. This corresponds to an internal degree of freedom that does not have any effect on the trajectory. The figures show some slight oscillations after 20 iterations, which are due to the fact that the arm is moving in the proximity of the target.

## 4. Discussion

Our case study shows that the active inference scheme can control the seven DoFs arm of a simulated PR2 robot—focusing here on the task of reaching a desired goal location from a (predefined) start location.

Our results illustrate that action control is accurate with intact proprioception and vision, and only partly impaired if noise is added to either of these modalities. The comparison of the trajectories of figure 2*b*,*c* shows that adding noise to proprioception is more problematic. The analysis of the dynamics of internal system variables (figures 4–7) helps us understanding the above results, highlighting the differential roles of proprioception and vision in this scheme. In the noisy proprioception scenario (figure 5), hidden states are significantly more uncertain compared with the reference case with no noise (figure 4). Yet, despite the uncertainty about joint angles, the robot can still rely on (intact) vision to infer where the arm is in space, and thus it is able to reach the target ultimately—although it follows largely suboptimal trajectories (in relation to its prior beliefs preferences). Multimodal integration or compensation is impossible if both vision and proprioception are sufficiently degraded (figure 7). In the noisy vision scenario, figure 6, noise has some effect on inferred causes but only affects hidden states (and ultimately action selection) to a minor extent.

This pattern of results shows that control is quintessentially multimodal and based on both vision and proprioception, and adding noise to either modality can be (partially) compensated for by appealing to the other, more precise dimension. However, proprioception and vision play differential roles in this scheme. Proprioception has a direct effect on hidden states and action selection; this is because action dynamics depend on reflex arcs that suppress proprioceptive (not visual) prediction errors (see §2.4). If the robot has poor proprioceptive information, then it can use multimodal integration and the visual modality to compensate and restore efficient control. However, if both modalities are degraded with noise, then multimodal integration becomes imprecise, and the robot cannot reduce error accurately—at least in the simplified control scheme assumed here, which (on purpose) does not include any additional corrective mechanism. Adding noise to vision is less problematic, given that in the (reaching) task considered here, it plays a more ancillary role. Indeed, our reaching task does not pose strong demands on the estimation of hidden causes for accurate control; the situation may be different if one requires, for example, to estimate the pose of a to-be-grasped object.

The above-mentioned results are consistent with a large body of studies showing the importance of proprioception for control tasks. Patients with impaired proprioception can still execute motor tasks such as reaching, grasping and locomotion, but their performance is suboptimal [35–38]. In principle, the scheme proposed here may be used to explain human data under impaired proprioception [4] or other deficits in motor control—or even to help design rehabilitation therapies. In this perspective, an interesting issue that we have not addressed pertains to the attenuation of proprioceptive prediction errors during movement. Heuristically, this sensory attenuation is necessary to allow the prior beliefs of the generative model to supervene over the sensory evidence that movement has not yet occurred (or in other words, to prevent internal states encoding the fact that there is no movement). This speaks to a dynamic gain control that mediates the attenuation of the precision of prediction errors during movement. In the example shown above, we simply reduced the precision of ascending proprioceptive prediction errors to enable movement. Had we increased their precision, the ensuing failure of sensory attenuation would have subverted movement; perhaps in a similar way to the poverty of movements seen in Parkinson's disease—a disease that degrades motor performance profoundly [39]. This aspect of precision or gain control suggests that being able to implement active inference in robots will allow us to perform simulated psychophysical experiments to illustrate sensory attenuation and its impairments. Furthermore, it suggests a robotic model of Parkinson's disease is in reach, providing an interesting opportunity for simulating pathophysiology.

Clearly, some of our choices when specifying the generative model are heuristic—or appeal to established notions. For example, adding a derivative term to equation (2.14) could change the dynamics in an interesting way. In general, the framework shown above accommodates questions about alternative models and dynamics through Bayesian model comparison. In principle, we have an objective function (variational free energy) that scores the quality of any generative model entertained by a robot—in relation to its embodied exchange with the environment. This means we could change the generative model and assess the quality of the ensuing behaviour using variational free energy—and select the best generative model in exactly the same way that people characterize experimental data by comparing the evidence for different models in Bayesian model comparison. We hope to explore this in future work.

We next hope to port the scheme to a real robot. This will be particularly interesting, because there are several facets of active inference that are more easily demonstrated in a real-world artefact. These aspects include a robustness to exogenous perturbations. For example, the movement trajectory should gracefully recover from any exogenous forces applied to the arm during movement. Furthermore, theoretically, the active inference scheme is also robust to differences between the true motor plant and the various kinematic constants in the generative model. This robustness follows from the fact that the movement is driven by (fictive) forces whose fixed points do not change with exogenous perturbations—or many parameters of the generative model (or process). Another interesting advantage of real-world implementations will be the opportunity to examine robustness to sensorimotor delays. Although not necessarily a problem from a purely robotics perspective, biological robots suffer non-trivial delays in the signalling of ascending sensory signals and descending motor predictions. In principle, these delays can be absorbed into the generative model—as has been illustrated in the context of oculomotor control [40]. At present, these proposals for how the brain copes with sensorimotor delays in oculomotor tracking remain hypothetical. It would be extremely useful to see if they could be tested in a robotics setting.

As noted above, active inference shares many similarities with the passive movement paradigm (PMP, [33,34]). Although strictly speaking, active inference is a corollary of the free-energy principle, it inherits the philosophy of the PMP in the following sense. Active inference is equipped with a generative model that maps from causes to consequences. In the setting of motor control, the causes are forces that have some desired fixed point or orbit. It is then a simple matter to predict the sensory consequences of those forces—as sensed by proprioception or robotic sensors. These sensory predictions can then be realized through open loop control (e.g. peripheral servos or reflex arcs); thereby realizing the desired fixed point (cf. the equilibrium point hypothesis [32]). However, unlike the equilibrium point hypothesis, active inference is open loop. This is because its motor predictions are informed by deep generative models that are sensitive to input from all modalities (including proprioception). The fact that action realizes the (sensory) consequences of (prior) causes explains why there is no need for an inverse model.

Optimal motor control formulations [15,16] are fundamentally different. Briefly, optimal control operates by minimizing some cost function in order to compute motor commands for a robot performing a particular motor task. Optimal control theory requires a mechanism for state estimation as well as two internal models: an inverse and forward model. This scheme also assumes that the appropriate optimality equation can be solved [41]. In contrast, active inference uses prior beliefs about the movement (in an extrinsic frame of reference) instead of optimal control signals for movements (in an intrinsic frame of reference). In active inference, there is no inverse model or cost function and the resulting trajectories are Bayes optimal. This contrasts with optimal control, which calls on the inverse model to finesse problems incurred by sensorimotor noise and delays. Inverse models are not required in active inference, because the robot's generative (or forward) model is *inverted* during the inference. Active inference also dispenses with cost functions, as these are replaced by the robot's (prior) beliefs (of note, there is a general duality between control and inference [15,16]). In brief, replacing the cost function with prior beliefs means that minimizing cost corresponds to maximizing the marginal likelihood of a generative model [42–44]. A formal correspondence between cost functions and prior beliefs can be established with the complete class theorem [45,46], according to which there is at least one prior belief and cost function that can produce a Bayes-optimal motor behaviour. In sum, optimal control formulations start with a desired endpoint (consequence) and tried to reverse engineer the forces (causes) that produce the desired consequences. It is this construction that poses a difficult inverse problem with solutions that are not generally robust—and are often problematic in robot control. Active inference finesses this problem by starting with the *causes* of movement, as opposed to the *consequences*.

Accordingly, one can see the solutions offered under optimal control as special cases of the solutions available under an (active) inferential scheme. This is because some policies cannot be specified using cost functions but can be described using priors; specifically, this is the case of solenoidal movements, whose cost is equal for every part of the trajectory [47]. This comes from variational calculus, which says that a trajectory or a policy has several components: a curl-free component that changes value and a divergence-free component that does not change value. The divergence-free motion can be only specified by a prior and not by a cost function. Discussing the relative benefits of control schemes with or without cost functions and inverse models is beyond the scope of this article. Here, it suffice to say that inverse models are generally hard to learn for robots, and cost functions sometimes need to be defined in *ad hoc* manner for robot control tasks. By eluding these constraints, active inference may offer a promising alternative to optimal control schemes. For a more detailed discussion on the links between optimal control and active inference, see [47].

Although active inference resolves many problems that attend optimal control schemes, there is no free lunch. In active inference, all the heavy lifting is done by the generative model—and in particular, the priors that define desired setpoints or orbits. The basic idea is to induce these attractors by specifying appropriate equations of motion within the generative model of the robot. This means that the art of generating realistic and purposeful behaviour reduces to creating equations of motion that have desired attractors. These can be simple fixed-point attractors as in the example above. They could also be much more complicated, producing quasi-periodic motion (as in walking) or fluent sequences of movements specified by heteroclinic cycles. All the more interesting theoretical examples in the theoretical literature to date rest upon some form of itinerant dynamics inherent in the generative model that sometimes have deep structure. A nice example of this is the handwriting example in [48] that used Lotka–Volterra equations to specify a sequence of saddle points—producing a series of movements. Simpler examples could use both attracting and repelling fixed points that correspond to contact points and collision points, respectively, to address more practical issues in robotics. Irrespective of the particular repertoire of attractors implicit in the generative model, the hierarchical aspect of the generative models that underlie active inference enables the composition of movements, sequences of movements and sequences of sequences [49–51]. In other words, provided one can write down (or learn) a deep generative model with itinerant dynamics, there is a possibility of simulating realistic movements that inherit deep temporal structure and context sensitivity.

In conclusion, in this article, we have presented a proof-of-concept implementation of robot control using active inference, a biologically motivated scheme that is gaining prominence in computational and systems neuroscience. The results discussed here demonstrate the feasibility of the scheme; having said this, further work is necessary to fully demonstrate how this scheme works in more challenging domains or whether it has advantages (from both technological and biological viewpoints) over alternative control schemes. Future work will address an implementation of the above scheme on a real robot with the same degrees of freedom as the PR2. Other predictive models could be developed, the generative model illustrated above is very simple and does not take advantage of the internal degrees of freedom. A key generalization will be integrating planning mechanisms that may allow, for example, the robot to proactively avoid obstacles or collisions during movement—or more generally, to consider future (predicted) and not only currently sensed contingencies [17,52–56]. Planning mechanisms have been described under the active inference scheme and can solve challenging problems such as the mountain–car problem [5], and can thus been seamlessly integrated in the model presented here—speaking to the scalability of the active inference scheme. Finally, one reason for using a biologically realistic model such as active inference is that it may be possible to directly map internal dynamics generated by the robot simulator (e.g. of hidden states) to brain signals (e.g. EEG signals reflecting predictions and prediction errors) generated during equivalent action planning or performance.

## Data accessibility

All data underlying the findings described in the manuscript can be downloaded from https://github.com/LPioL/activeinference_ROS.
